# Synergic effect of simvastatin in combination with amphotericin B against environmental strains of *Cryptococcus neoformans* from northeastern Brazil: a prospective experimental study

**DOI:** 10.1590/1516-3180.2019.0107.R2.16092019

**Published:** 2020-04-22

**Authors:** Tássio Henrique Sousa Silva, Claudiane Vansoski Araújo, Khelvin Myner da Costa Santos, Nathanael dos Santos Alves, Thayse Haylene Soares Gomes, Andressa Kelly Ferreira e Silva, Nayra Cristina Lira dos Santos Silva, Evandro César Bezerra Damasceno, Andressa Maria Aguiar de Carvalho, Maria Gabriela Araújo Mendes, Henrique Barros Caminha, Tatiane Caroline Daboit, Thatiana Bragine Ferreira, Leonardo Eurípedes Andrade-Silva, Mario León Silva-Vergara, Kennio Ferreira-Paim, Fernanda Machado Fonseca

**Affiliations:** I BSc. Biomedic, Department of Biomedicine, Universidade Federal do Piauí, Parnaíba (PI), Brazil.; II BSc. Biomedic, Department of Biomedicine, Universidade Federal do Piauí, Parnaíba (PI), Brazil.; III BSc. Biomedic, Department of Biomedicine, Universidade Federal do Piauí, Parnaíba (PI), Brazil.; IV BSc. Biomedic, Department of Biomedicine, Universidade Federal do Piauí, Parnaíba (PI), Brazil.; V BSc. Biomedic, Department of Biomedicine, Universidade Federal do Piauí, Parnaíba (PI), Brazil.; VI BSc. Biomedic, Department of Biomedicine, Universidade Federal do Piauí, Parnaíba (PI), Brazil.; VII BSc. Biomedic, Department of Biomedicine, Universidade Federal do Piauí, Parnaíba (PI), Brazil.; VIII BSc. Biomedic, Department of Biomedicine, Universidade Federal do Piauí, Parnaíba (PI), Brazil.; IX MSc. Doctoral Student, Department of Biomedical Sciences, Universidade Federal do Piauí, Parnaíba (PI), Brazil.; X MSc. Doctoral Student, Department of Biomedical Sciences, Universidade Federal do Piauí, Parnaíba (PI), Brazil.; XI MSc. Doctoral Student, Department of Biomedical Sciences, Universidade Federal do Piauí, Parnaíba (PI), Brazil.; XII PhD. Associate Professor, Department of Medicine, Universidade Federal do Piauí, Parnaíba (PI), Brazil.; XIII MSc. Doctoral Student, Department of Infectious and Parasitic Diseases, Universidade Federal do Triângulo Mineiro, Uberaba (MG), Brazil.; XIV PhD. Biomedic, Department of Clinical Pathology, Universidade Federal do Triângulo Mineiro, Uberaba (MG), Brazil.; XV PhD. Associate Professor, Department of Infectious and Parasitic Diseases, Universidade Federal do Triângulo Mineiro, Uberaba (MG), Brazil.; XVI PhD. Associate Professor, Department of Microbiology, Universidade Federal do Triângulo Mineiro, Uberaba (MG), Brazil.; XVII PhD. Associate Professor, Department of Biomedicine, Universidade Federal do Triângulo Mineiro, Uberaba (MG), Brazil.

**Keywords:** Cryptococcosis, Cryptococcus neoformans, Drug resistance, fungal, Antifungal agents, Drug interactions, Cryptococcosis treatment, HIV infection, Fractional inhibitory concentration index, Antifungal therapy

## Abstract

**BACKGROUND:**

Statins are used as cholesterol-lowering drugs and may also have direct antimicrobial effects.

**OBJECTIVE:**

To evaluate synergic interactions between simvastatin and both amphotericin B and fluconazole, against environmental strains of *Cryptococcus neoformans* isolated from captive birds’ droppings.

**DESIGNAND SETTING:**

Experimental study conducted at Federal University of Piauí, Parnaíba, in collaboration with Federal University of Triângulo Mineiro, Uberaba, Brazil.

**METHODS:**

Statin susceptibility tests of *Cryptococcus neoformans* samples were performed as prescribed in standards. Interactions of simvastatin with amphotericin and fluconazole were evaluated using the checkerboard microdilution method. Presence of these interactions was quantitatively detected through determining the fractional inhibitory concentration index (FICI).

**RESULTS:**

Isolates of *Cryptococcus neoformans* were obtained from 30 of the 206 samples of dry bird excreta (14.5%) that were collected from pet shops and houses. Ten isolates were selected for susceptibility tests. All of them were susceptible to amphotericin and fluconazole. All presented minimum inhibitory concentration (MIC) > 128 µg/ml and, thus, were resistant *in vitro* to simvastatin. An *in vitro* synergic effect was shown through combined testing of amphotericin B and simvastatin, such that six isolates (60%) presented FICI < 0.500. Two isolates showed considerable reductions in MIC, from 1 µg/ml to 0.250 µg/ml. No synergic effect was observed through combining fluconazole and simvastatin.

**CONCLUSION:**

These results demonstrate that simvastatin should be considered to be a therapeutic alternative, capable of potentiating the action of amphotericin B. However, further studies are necessary to clarify the real effect of simvastatin as an antifungal agent.

## INTRODUCTION

*Cryptococcus neoformans* is an encapsulated and opportunistic yeast fungus that has worldwide distribution. In the urban environment, it is often found in soil contaminated with dried bird excreta, and thus can infect humans and other animals through inhalation.^1^^,^^2^

Currently, the treatment for cryptococcosis is based on use of amphotericin B alone or in combination with 5-flucytosine or azoles, in the early stages of the disease. However, the high toxicity associated with these antifungals may restrict their use in special clinical settings, such as in cases of chronic kidney diseases. In situations of secondary prophylaxis, use of fluconazole is indicated to minimize the risk of recurrence of infection in patients who are still in an immunosuppressive state.^3^ Treatment of cryptococcosis using azoles (e.g. fluconazole) requires long periods and can favor the emergence of microbial resistance. Although azoles are less toxic than polyenes, they present low efficacy for the initial treatment of the disease. In contrast, amphotericin B provides a more effective therapeutic response against the fungus, although its use should be limited because of its toxicity.^2^

Emergence of antifungal resistance among environmental and clinical isolates of *Cryptococcus neoformans* has been described over the last few decades. Prolonged use of antifungals for treating patients with immunosuppressive disorders such as acquired immune deficiency syndrome (AIDS) may contribute towards antifungal resistance.^4^^,^^5^^,^^6^

Development of new therapeutic strategies is therefore extremely important, given the low number of antifungals available and the scarcity of these drugs in limited-resource settings. In this context, new drugs can contribute towards treatment of cryptococcosis through enhancing the effect of traditional antifungals.^7^

Statins are drugs that are used to treat cardiovascular diseases relating to high cholesterol levels in humans. The mechanism of action of statins is based on inhibition of 3-hydroxy-3-methyl-glutaryl-CoA reductase (HMG-CoA), an enzyme responsible for liver cholesterol biosynthesis. As this enzyme becomes inhibited by statins, the pathway of cholesterol synthesis is blocked, thus resulting in a decrease in LDL cholesterol levels. Additionally, this enzyme synthesizes mevalonic acid, an important precursor in the synthesis of sterols such as ergosterol in fungi and cholesterol in humans.^8^

Hence, statins are important inhibitors of precursors of the synthesis of these sterols. Several studies have demonstrated that some statins present antifungal activity: for example, fluvastatin and simvastatin against species of *Candida* and *Cryptococcus*. Therefore, combining statins with antifungal agents could decrease the length of time for which infected patients are exposed to toxic drugs. Consequently, this would reduce the side effects from use of antifungal agents, especially in cases of emergence of fungal strains that are resistant to conventional treatment.^9^^,^^10^

In this context, given the increasing numbers of cases of antifungal resistance and the toxicity presented by the conventional treatment scheme, the antifungal potential of simvastatin alone and in association with amphotericin B and fluconazole was tested on *Cryptococcus* isolates that were recovered from the dried feces of captive birds in northeastern Brazil.

## METHODS

### Ethics statement

This study was approved by the Research Ethics Committee of the Federal University of Piauí (Universidade Federal do Piauí, UFPI), Campus Ministro Reis Velloso, Parnaíba, Piauí, Brazil, under the number CAAE 45296315.5.0000.5669, on July 10, 2015.

### Environmental samples

Between September 2015 and April 2017, a total of 206 dry fecal samples from captive birds of the species *Columbia livia, Melopsittacus undulatus* and *Lonchura striata* were collected from pet shops and houses located in different districts of Parnaíba, Piauí, Brazil. This number of samples comprised the total number available for collection at the time of the study. The dried feces were picked up from the birdcages using sterile swabs and were inoculated into tubes containing 10 ml of sterile saline solution with 0.4 g/l of chloramphenicol. It was not possible to determine how long these samples had been exposed before the cleaning procedures were performed.

Isolates of *Cryptococcus neoformans* were obtained from 30 of the 206 samples of dry bird excreta (14.5%) that had been collected from pet shops and houses. Ten of these isolates were selected for susceptibility tests because all the 30 isolates belonged to the same molecular type.

Each of the 206 feces samples was placed in a separate tube (thus, there were 206 tubes). The tubes were shaken to mix the contents for two minutes and the samples were then left to settle at room temperature for 30 minutes. Next, 100 ml of the supernatant was inoculated onto Niger seed (*Guizotia abyssinica*) agar plates supplemented with 0.4 g/ml of chloramphenicol. Each sample was spread on four plates, incubated at 35 °C and examined daily for 10 days, macroscopically, in order to identify any presence of smooth, beige to dark brown colonies suggestive of *Cryptococcus* spp.^11^

These colonies were streaked onto Sabouraud agar at 35 °C for 48 hours and were identified using the India ink test and urease. Canavanine-glycine-bromothymol blue (CGB) medium was used to differentiate *Cryptococcus neoformans* from *Cryptococcus gattii*. The isolates thus obtained (which were clones) were then stored until the tests were performed. To ensure preservation, some of the cell masses were stored in distilled water at 4 °C, while others were stored in yeast peptone dextrose broth (Difco Laboratories, Sparks, MD, USA) with 30% glycerol at 4 °C.

### Deoxyribonucleic acid extraction

Deoxyribonucleic acid (DNA) extraction was performed as previously described by Bolano et al.^12^ Briefly, a single colony was spread on yeast malt agar (Difco Laboratories) at 37 °C for 72 hours. Then, a loopful of cells from the culture was transferred to Eppendorf tubes and incubated at -20 °C overnight.

Next, 500 ml of cell lysis buffer [1.25 mol/l NaCl, 0.5% sodium dodecyl sulfate (SDS), 100 mmol/l Tris-hydrochloride (Tris-HCl) at pH 7.5 and 0.25 mol/l ethylenediamine tetraacetic acid (EDTA) at pH 8.0] and 5 ml of 2-mercaptoethanol (Sigma, Steinheim, Germany) were added. The tubes were vortexed for 2 minutes and incubated at 65 °C for 1 hour. Then, 500 ml of phenol-chloroform-isoamyl alcohol (25:24:1) was added, shaken for 2 minutes at room temperature and centrifuged at 8150 g, at 4 °C for 15 minutes.

To precipitate the genomic DNA, the aqueous phase was transferred to a new tube and 500 ml of isopropanol alcohol was added and this mixture was incubated at -20 °C overnight. The solution was centrifuged at 4 °C for 15 minutes at 8150 g to pellet the DNA and was then washed with ice-cold 70% ethanol. It was again centrifuged as above and was then air-dried.

The DNA was resuspended in 500 ml of Tris-EDTA (TE) buffer (10 mmol/l Tris-HCl at pH 7.5 and 0.5 mol/l EDTA at pH 8.0) containing 50 mg/ml of ribonuclease-A (RNAse-A) (Invitrogen, Carlsbad, CA, USA). It was then incubated at 37 °C for 30 minutes and stored at 4 °C for polymerase chain reactions (PCR) to be performed.

### Restriction fragment length polymorphism (RFLP) of *URA5* gene

Amplification of the *URA5* gene was carried out in a final volume of 25 µl. Each reaction contained 10 µg of DNA, 2.5 µl of 1x PCR buffer (10 mmol/l Tris-HCl at pH 8.3, 50 mmol/l KCl and 1.5 mmol/l MgCl_2_), 0.2 mmol/l each of deoxyadenosine triphosphate (dATP), deoxycytidine triphosphate (dCTP), deoxyguanosine triphosphate (dGTP) and deoxythymidine triphosphate (dTTP), 1.25 U of Taq DNA polymerase (Invitrogen, São Paulo, Brazil) and 25 ng of each primer: *URA5* (5’-ATGTCCTCCCAAGCCCTCGACTCCG-3’) and *SJ01* (5’-TTAAGACCTCTGAACACCGTACTC-3’).

From this, a fragment of approximately 800 bp was produced. Thirty-four PCR cycles were performed in a PTC-100 thermocycler (MJ Research Inc., Watertown, MA, USA), consisting of initial denaturation at 94 °C for 4 minutes, followed by denaturation at 94 °C for 45 seconds, annealing at 57 °C for 1 minute, extension at 72 °C for 1 minute and final extension cycle at 72 °C for 10 minutes.

The amplified products were mixed with an equal volume of 2x loading buffer (0.5% bromophenol blue, 0.5% xylene-cyanol and 60% glycerol) and then separated by means of electrophoresis on 1.5% agarose gel (Invitrogen, Barcelona, Spain) in 1x Tris-acetate-EDTA (TAE) buffer at 90 V for 2 hours. They were then stained with 0.5 mg/ml ethidium bromide and viewed under UV light.

Subsequently, 15 µl of PCR products were double-digested using *Sau*96I (10 U/µl) and *Hha*I (20 U/µl) at 37 °C for 3 hours, and then separated by means of electrophoresis on 3% agarose gel at 90 V for 3 hours. RFLP patterns were assigned visually by comparison with the patterns obtained from the standard reference strains (VNI, WM-148; VNII, WM-626; VNIII, WM-628; VNIV, WM-629; VGI, WM-179; VGII, WM-178; VGIII, WM-175; and VGIV, WM-779).^13^ In all the reactions, the reference strain of *C. neoformans* ATCC 90112 (American Type Culture Collection, Manassas, VA, USA) was included as a positive control.

### Minimum inhibitory concentration (MIC) and fractional inhibitory concentration index (FICI)

The broth microdilution test was performed in accordance with the prescriptions of the Clinical and Laboratory Standards Institute (CLSI). The incubation temperature was changed from 37 °C to 33 °C in order to standardize the growth of the strains.

Fluconazole (Zoltec, Pfizer, Guarulhos, SP, Brazil) was initially dissolved in sterile water in accordance with document M27A3 of the CLSI.^14^ Amphotericin B (Sigma, São Paulo, Brazil) was diluted in dimethyl sulfoxide (DMSO) (Vetec, Brazil). The serial dilutions of the antifungal agents were prepared in RPMI 1640 medium (with L-glutamine and without sodium bicarbonate) (MP Biomedicals, France), and were buffered to pH 7.0 with 0.165 mol/l morpholinopropane sulfonic acid (MOPS) (Êxodo Scientific, Brazil). The fluconazole concentration ranged from 0.0625 to 64 μg/ml and the final concentrations of amphotericin B ranged from 0.0312 to 16 μg/ml. The *Candida krusei* strain ATCC 6258 were used as a quality control.

The minimum inhibitory concentration (MIC) results were defined for *C. neoformans* as specified by the CLSI^14^ and by other authors, as follows: MIC > 64 μg/ml was deemed to be resistant; MIC between 16 and 32 μg/ml was considered to be susceptible dose-dependent (SDD); and MIC < 8 μg/ml was considered to be susceptible.^15^^,^^16^

The interactions of simvastatin (Pharmanostra Ltda., São Paulo, Brazil) with amphotericin and of simvastatin with fluconazole were evaluated by means of the checkerboard microdilution method.^17^ The stock solution of simvastatin was prepared with DMSO at a concentration of 12800 μg/ml and was then diluted in RPMI 1640 medium buffered with MOPS in the proportions of 1:50. The final concentrations in the wells ranged from 128 to 0.25 μg/ml. The interactions between these drugs were evaluated quantitatively by determining the fractional inhibitory concentration index (FICI). They were classified as synergistic if FICI ≤ 0.500, indifferent if FICI > 0.500-4.0 and antagonistic if FICI > 4.0.^17^^,^^18^ All the tests were performed in duplicate and on different days.

## RESULTS

**Figure 1.**
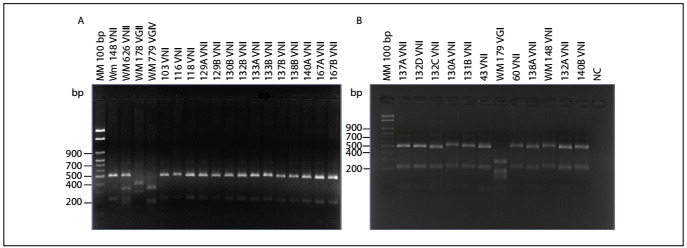
A: Representative agarose gel electrophoresis of *URA5* polymerase chain reaction-restriction fragment length polymorphism showing the identification of molecular types VNI, VNII, VGII and VGIV (control samples) and the environmental *Cryptococcus neoformans* isolates of this study, all of genotype VNI (columns 6 to 19). MM: 100 bp molecular marker (column 1). B: Representative agarose gel electrophoresis of *URA5* polymerase chain reaction-restriction fragment length polymorphism showing the identification of molecular types VNI and VGI (control samples) and the environmental *Cryptococcus neoformans* isolates of this study, all of genotype VNI (columns 2 to 7; 9 and 10; 12 and 13). MM: 100 bp molecular marker (column 1). NC = negative control.

Isolates of *Cryptococcus* spp. were recovered from 30 of the 206 samples of dried feces from captive birds that were collected from pet shops (14.5%). According to the bird species, the isolates of *Cryptococcus* spp. were recovered as follows: 28 (93.3%) from *Columbia livia*, one (3.3%) from *Melopsittacus undulates* and one (3.3%) from *Lonchura striata domestica*. All of the isolates presented capsules, urease production, growth at 37 °C and melanin production on Niger seed agar. In addition, all of them presented a negative canavanine glycine bromothymol blue (CGB) test. According to the molecular typing, all the isolates of *Cryptococcus neoformans* were identified as VNI (Figure 1).

Ten isolates were selected to be subjected to the susceptibility tests. All of them presented susceptibility to amphotericin and fluconazole. On the other hand, all of these isolates presented MIC > 128 µg/ml in relation to simvastatin and, thus, were considered resistant *in vitro* to this drug.

In the test on amphotericin B and simvastatin in combination, 6 (60%) of the 10 isolates studied presented FICI < 0.500, thus revealing a synergistic *in vitro* effect. Moreover, 3 samples (30%) presented FICI values of 0.501, i.e. only slightly above the cutoff value for synergism. Synergism between simvastatin and amphotericin B was detected at a simvastatin concentration of 0.250 μg/ml. The isolates PI1543 and PI16202 showed MIC reductions from 1 µg/ml to 0.250 µg/ml, thus indicating clearly the synergistic effect of combining simvastatin with amphotericin B. However, when simvastatin was combined with fluconazole, no synergistic effect was observed (Table 1).

**Table 1.** Minimum inhibitory concentration (MIC), mean fractional inhibitory concentration index (FICI) and interactions of simvastatin (SIM) and amphotericin B (AMB) and fluconazole (FLZ) against environmental strains of *Cryptococcus neoformans*

Strain	Minimum inhibitory concentration (MIC)											
	AMB (µg/ml)	AMB + SIM µg/ml)	SIM (µg/ml)	SIM + AMB (µg/ml)	FICI (AMB + SIM)	Interaction	FLZ (µg/ml)	FLZ + SIM (µg/ml)	SIM (µg/ml)	SIM + FLZ (µg/ml)	FICI (SIM + FLZ)	Interaction
*Candida krusei* ATCC 6258	1.0	N/A	> 128	N/A	N/A	N/A	32	N/A	> 128	N/A	N/A	N/A
PI1543	1.0	0.250	> 128	0.250	0.251	Synergism	8	64	> 128	> 128	9	Antagonism
PI1544	0.25	0.125	> 128	0.250	0.501	Indifferent	8	64	> 128	> 128	9	Antagonism
PI16167	0.5	0.125	> 128	0.250	0.251	Synergism	8	64	> 128	0.25	8	Antagonism
PI16180	1.0	0.500	> 128	0.250	0.501	Indifferent	4	32	> 128	> 128	9	Antagonism
PI16198	0.5	0.125	> 128	0.250	0.251	Synergism	8	64	> 128	> 128	9	Antagonism
PI16199	0.5	0.500	> 128	0.250	1.000	Indifferent	8	64	> 128	0.25	8	Antagonism
PI16200	0.5	0.250	> 128	0.250	0.501	Indifferent	8	64	> 128	> 128	9	Antagonism
PI16202	1.0	0.250	> 128	0.250	0.251	Synergism	8	8	> 128	1	1	Indifferent
PI17123	0.5	0.125	> 128	0.250	0.251	Synergism	8	64	> 128	> 128	9	Antagonism
PI17213	0.5	0.125	> 128	0.250	0.251	Synergism	8	64	> 128	> 128	9	Antagonism

N/A = not applicable; AMB = amphotericin B; SIM = simvastatin; FLZ = fluconazole; FICI = fractional inhibitory concentration index.

## DISCUSSION

In the present study, *Cryptococcus neoformans* was isolated from 14.5% of the samples collected. Similar results were found in some other regions of Brazil: *Cryptococcus neoformans* was isolated in 17.3% of the captive birds droppings in a study in the city of Uberaba, Minas Gerais, and in 18.5% in the city of Salvador, Bahia.^19^^,^^20^ However, in other regions of Brazil, the prevalence has differed, ranging 25.3% in the southern region to 50% in the central region of this country.^21^ The variation in the isolation rates can be related to climatic factors, since high temperatures inhibit fungal growth.^22^ Additionally, the methodology used and conditions of bird rearing may interfere with the isolation rates.^21^

*Cryptococcus neoformans* and *Cryptococcus gattii* have at least eight different molecular types that present variable geographical distribution within the eco-epidemiological context of the disease in Brazil and worldwide. The isolates of the present study were identified as the VNI genotype. This result was concordant with what has previously been found in Brazil and other parts of the world: in previous studies, the VNI genotype was detected in 95% of the environmental samples in which *Cryptococcus neoformans* was identified.^19^ The predominance of the VNI genotype among the isolates recovered also enables better interpretation of the antifungal susceptibility test, given the consequent homogeneity of the population. On the other hand, it is well known that certain genotypes, such as VNIV, can frequently be recovered from regions such as around the Mediterranean basin.^23^

The susceptibility profile of *Cryptococcus* spp. relating to antifungals was evaluated in different countries between 1990 and 2004. The resistance to amphotericin B, 5-flucytosine and fluconazole that was detected was less than 1%. Isolates from North America presented MIC ≤ 8 µg/ml in relation to fluconazole, while in other geographical regions (Latin America and Africa), the strains showed susceptibility to fluconazole in 94% to 100% of the cases. Additionally, the isolates presented 99% susceptibility to amphotericin B, with MIC ≤ 1 µg/ml.^24^

There is increasing interest in evaluating the antifungal activity of antifungal drugs, especially in the field of combined therapy.^25^ In addition, evidence demonstrating the potential use of statins for preventing and treating infections has been reported. Statins have been shown to attenuate the pathogenicity of microorganisms through modulating the signaling and other regulatory pathways that are involved in the infection.^26^^,^^27^ The activity of statins against *Cryptococcus* and *Candida* species, with particular emphasis on simvastatin, used in isolation or in combination with classical antifungals such as amphotericin B and fluconazole, was previously described by Brilhante et al.^10^ Chin et al.^9^ demonstrated that the statin fluvastatin had fungicidal action against different species of *Candida*. Moreover, a synergistic effect was observed through combinations of fluvastatin with fluconazole and itraconazole, two commonly used azole compounds. With these combinations, both fluconazole and itraconazole exhibited potent activity against species of *Candida* and also against *Cryptococcus neoformans*.

The *in vitro* interactions of the effects of various statins, including simvastatin and various azole antifungals, against different opportunistic pathogenic fungi such as *Candida* and *Aspergillus* species, were investigated in another study. Fluconazole was found to act synergistically against *Aspergillus fumigatus* in combination with simvastatin, lovastatin and atorvastatin. The interaction of the combination of miconazole and simvastatin against *Candida glabrata* was not significant, but the sensitivities to this azole compound differed by one or two dilution steps between the isolates.^28^

The results from the present study demonstrated the efficacy of simvastatin as a synergistic agent in combination with amphotericin B against environmental isolates of *Cryptococcus neoformans*. However, no inhibition of yeast growth was observed when the effect of simvastatin was evaluated in isolation or in combination with fluconazole, and the MIC values found were elevated. Similar results were demonstrated by Brilhante et al.,^10^ such that all the strains of *Cryptococcus neoformans* evaluated were inhibited by the combination of simvastatin and amphotericin B. However, no such inhibition was observed in relation to the combination of simvastatin and fluconazole.

The antifungal effect observed when simvastatin was evaluated in combination with amphotericin B may have been due to the action of statins in the process of formation of ergosterol, which has similarity to human cholesterol. This drug acts by inhibiting 3-hydroxy-3-methyl-glutaryl-coenzyme-A (HMG-CoA) reductase in both humans and fungi, since both of these act on the same synthetic pathway as mevalonate. The *in vitro* activity of statins (fluvastatin, simvastatin, pravastatin and lovastatin) against strains of species of *Candida* and *Cryptococcus* was evaluated, and only fluvastatin demonstrated inhibition of these yeasts. However, *in vitro* interaction of fluvastatin with fluconazole and amphotericin B has been observed, which demonstrates the potential synergism between these drugs.^9^

It is important to note that, in the present study, two isolates had a MIC of 1.0 μg/ml for amphotericin B, which was the highest MIC among the strains evaluated. These isolates presented a considerable reduction in MIC (0.250 μg/ml) when the combination of amphotericin B and simvastatin was tested, which again emphasizes the potential use of this combination of these drugs. On the other hand, among the four isolates presenting indifferent results regarding the association between amphotericin B and simvastatin, three of them demonstrated values that were slightly above the cutoff that had been adopted.

Given that all of the isolates of the present study belonged to the same molecular type, it is possible that a greater number of samples tested would have enabled clear demonstration of the synergistic effects between these two drugs. However, we collected the number of samples that were available at the time when the study was performed (i.e. 206 samples). Our isolation rate of 14.5% was in line with what had been found in other studies using the same methodology.^19^^,^^20^ On the other hand, increasing the number of samples would also potentially have led to greater variability of the fungi in the samples (there are eight molecular types of *Cryptococcus*).

## CONCLUSION

The data of this study demonstrate that simvastatin should be considered to be a possible therapeutic alternative, with the capacity to potentiate the action of amphotericin B. Through using this drug, the duration of cryptococcosis treatment could potentially become shorter and, consequently, the time for which patients are exposed to the toxic effects of this antifungal could be reduced. In addition, statins may have an important role in the future, as a new treatment alternative in situations of resistance to antifungals.
